# On the Surface Hardening of Zinc Sulfide Windows by Gallium Sulfide †

**DOI:** 10.3390/ma17225622

**Published:** 2024-11-18

**Authors:** Hayat Soufiani, Alexandros Kostogiannes, Clara Rivero-Baleine, Kathleen A. Richardson, Romain Gaume

**Affiliations:** 1 CREOL, The College of Optics and Photonics, University of Central Florida, 4304 Scorpius St., Orlando, FL 32816, USA; alexandros.kostogiannes@ucf.edu (A.K.); kcr@creol.ucf.edu (K.A.R.); 2 Lockheed Martin Corporation, 5600 Sand Lake Rd., Orlando, FL 32819, USA; clara.rivero-baleine@creol.ucf.edu

**Keywords:** infrared windows, Ga_2_S_3_-ZnS system, mechanical properties of ceramics, transmission, doping profile

## Abstract

This study examines the effect of gallium doping on the phase transformation, transmission, and hardness of commercial multispectral-grade ZnS specimens exposed to Ga_2_S_3_ vapor. Using secondary ion mass spectrometry, we show that Ga diffusion extends into the subsurface down to several tens of microns. X-ray diffraction patterns reveal minimal to no precipitation of wurtzite, resulting in limited infrared transmission loss after treatment. We report a monotonic increase in Vickers surface microhardness with increasing Ga concentration, reaching values more than double those of untreated windows. Future work will focus on optimizing this process and evaluating its effectiveness in enhancing the durability of ZnS windows under harsh environmental conditions.

## 1. Introduction

Multispectral zinc sulfide (MS-ZnS) is a broadband optical ceramic window extensively used in the aviation and defense sectors. These types of applications require both high durability and transparency to maintain window integrity during operations. Pure ZnS exhibits polymorphism as it crystallizes into cubic (sphalerite), hexagonal (wurtzite), and numerous polytypic forms, referred to as hexagonality [1,2]. Due to the isotropy of its refractive index, sphalerite can be formed into scatter-free transparent ceramics suitable for optical imaging. Three distinct processes have been employed in the production of polycrystalline ZnS: vacuum sublimation, hot pressing (HP), and chemical vapor deposition (CVD). CVD-ZnS was found to have superior purity, enhanced density, and better optical homogeneity. In addition, yellow-colored CVD-ZnS can be further treated by hot isostatic pressing (HIP) to obtain a water-clear material, called multispectral ZnS (MS-ZnS), with an increased transmission over the 0.4 to 14 µm spectral range [2,3]. Although HIPing enhances transmission by stabilizing sphalerite and reducing porosity and hexagonality, it also causes grains to grow from a few microns to 20–200 µm [3,4], resulting in reduced mechanical strength [4,5,6] and overall performance [6,7,8,9]. Hence, multiple approaches have been used to strengthen CVD- and MS-ZnS through both surface and bulk modifications.

Surface treatments included film deposition [6,10,11], annealing under different atmospheres [6,10], and ion implantation [6,12,13]. However, most of these techniques have been discontinued because of unsatisfactory hardness enhancement, sample cracking, or transmission deterioration.

Bulk treatment techniques relying on solute strengthening [6,10,14], grain size refinement [6], ZnS-based composite fabrication via powder sintering [15,16], precipitation hardening [14], or development of glass ceramics [17] have also been considered. While solute strengthening and grain size refinement did not yield substantial improvements in the mechanical properties, ZnS-based composites showed promising results in terms of toughness, Young’s modulus, and hardness. Notably, when examining sintered Ga_2_S_3_-ZnS ceramics, Dunn et al. [14] revealed a correlation between increasing Ga_2_S_3_ content and decreasing grain size, resulting in substantial enhancements to both hardness and fracture toughness. The highest Vickers hardness of 427 kg·mm^−2^ (4.18 GPa) was achieved for a composition containing 12 mol. % Ga_2_S_3_ with a grain size of 6 µm. The group stated that several Ga_2_S_3_-ZnS solid solutions exhibited transmission properties comparable to that of pure ZnS; however, no experimental IR spectra were reported. Similarly, glass ceramics within the Ga-La-S-ZnS system have been explored as potential durable materials for long-wave infrared (LWIR) optics [17]. Studies showed that the ZnGa_2_S_4_ crystallites embedded within the glass matrix increased hardness significantly, achieving values from 2.8 to 5.6 GPa, compared to 1.5 GPa for CVD-ZnS. However, the material was reported as opaque, likely due to factors such as extensive crystallization, oxygen contamination, and the presence of multiple phases [18].

The current study examines the effect of Ga diffusion on the microstructure, transmission, and hardness of MS-ZnS. To prevent oxidation, the process involved vacuum heat treatment of MS-ZnS blanks in the presence of Ga_2_S_3_ powder. The temperature and mass ratio of the reactants, $m_{{Ga}_{2}S_{3}}/m_{ZnS}$, were carefully selected to retain the sphalerite-pure material and avoid the precipitation of wurtzite or tetragonal zinc thiogallate ZnGa_2_S_4_ [14,19].

## 2. Experiment

**Figure 1:**
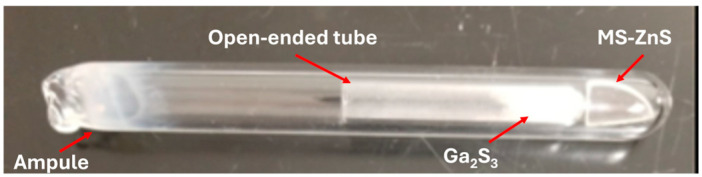
Ga_2_S_3_ powder enclosed in a small tube to avoid direct contact with MS-ZnS. Both reactants are enclosed in a vacuum-sealed quartz ampoule to prevent oxidation.

Discs of MS-ZnS Cleartran™ (American Photonics, Sarasota, FL, USA), 25 mm in diameter and 2.50 mm thick with an average grain size of 40 µm, and gallium sulfide (Ga_2_S_3_) powder (99.99% Thermo Fisher Scientific, Waltham, MA, USA) were used. This powder was loaded into a small open-ended quartz tube and placed next to an MS-ZnS window in a larger quartz ampoule (Figure 1). The reaction chamber was then sealed under vacuum (200 mTorr). As part of the process optimization, the sealed ampoules were held at constant temperatures in the range of 800–900 °C for 1–4 weeks. For comparison, three Ga_2_S_3_-treated MS-ZnS coupons labeled as Samples #1, #2, and #3 with increasing $m_{{Ga}_{2}S_{3}}/m_{ZnS}$ along with annealed MS-ZnS without Ga_2_S_3_ were prepared under the same conditions of time and temperature. All samples were polished on both sides using 800 and 1200 grit pads, and surface was finished with a cloth pad and 0.5 µm alumina suspension. Secondary ion mass spectrometry (SIMS) was performed with an Adept 1010 Dynamic system (Chigasaki, Japan) equipped with an $O_{2}^{+}$ ion source. For quantification and correction for any variations in instrument parameters, changes in ion collections, sputtering yields, and ionization efficiencies [20], the relative sensitivity factors (RSFs) were obtained using three standards made by implanting 0.1, 0.5, and 1 at. % of ^69^Ga ions into undoped MS-ZnS coupons at CuttingEdge ions LLC, Anaheim, CA, USA (see Appendix A). X-ray diffraction (XRD) analysis was carried out using a Panalytical diffractometer (Empyrean, Malvern, UK) equipped with a copper anticathode (Cu_α1_ = 1.5406 Å). Diffraction patterns were collected over the range 20° < 2θ < 90° and fitted using HighScore Plus (version 4.5) software for phase identification. LaB_6_ powder was used as an internal standard for all XRD analyses. A PerkinElmer fluorescence spectrometer LS 45 fitted to a Xe source was used to collect photoluminescence (PL) spectra across a wavelength range of 410–700 nm at room temperature. Infrared transmission was recorded over 400–7000 cm^−1^ using a Fourier Transform Infrared (FTIR) Nicolet iS5 (Wilmington, MA, USA). The hardness of the samples was measured using a DUH-221S Shimadzu dynamic ultra-microhardness tester (Kyoto, Japan) fitted with a Vickers diamond pyramid (Kyoto, Japan). The microhardness reported for each sample was the average of 100 measurements obtained at room temperature under a load of 100 mN applied at 5.00 mN/s and held for 10 s.

## 3. Results and Discussion

### 3.1. Microstructure and Ga Depth Profiling

**Figure 2:**
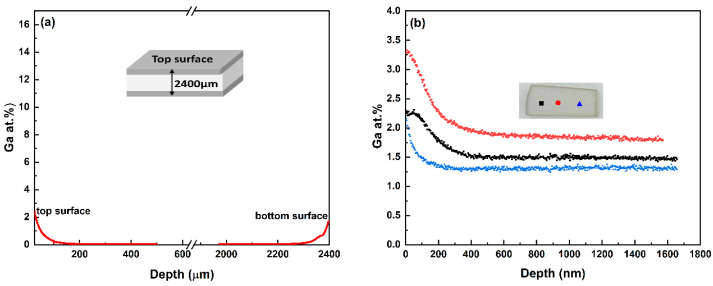
(**a**) Ga concentration profiles throughout the cross-section by SIMS 2D-image mode. (**b**) Ga depth profiles measured on the surface by SIMS depth profiling mode. These measurements were taken at three different locations 800 µm apart, as shown on the inset, and are labeled red, black and blue for clarity.

The cross-sectional concentration profiles of Ga for Sample #3 are depicted in Figure 2a, demonstrating that Ga_2_S_3_ has penetrated more deeply into the sample from both ends. The diffusion profile is nearly symmetrical with respect to the midplane of the sample as one might expect. Similarly, there is a slight non-uniformity in Ga doping across the surface of the same sample, as shown in Figure 2b, where the Ga depth profiles at three different locations are compared. Potential parameters that may play a role in Ga non-homogeneity are temperature irregularities, ZnS grain size uniformity, and orientation relative to the surface.

**Figure 3:**
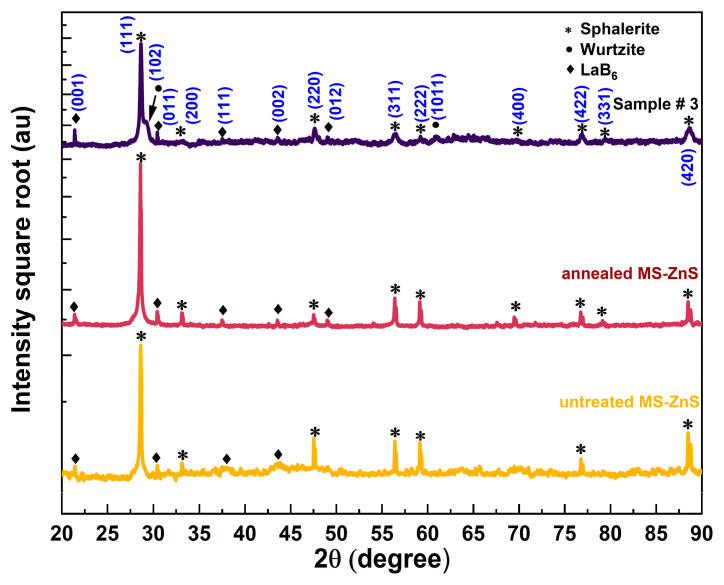
From bottom to top: X-ray diffraction patterns of untreated, annealed, and Sample #3 compared to *s* and *w* ZnS as per reference card #01-074-6110 and #01-089-2191, respectively, (see Appendix A for details).

X-ray diffraction patterns of the untreated, annealed, and Sample #3 coupons are presented in Figure 3. The square root of the intensities was plotted against 2θ to make peaks with smaller intensities more visible. The diffraction patterns exhibit similar characteristics, with minor variations in peak intensities and positions. As anticipated, the untreated MS-ZnS sample displays a pure cubic sphalerite (*s*) phase, characterized by an intense *s* (111) diffraction peak. Likewise, the heat-treated MS-ZnS coupon maintains the pure sphalerite structure, showing no transformation to the wurtzite (*w*) phase during annealing. This is evidenced by the absence of wurtzite diffraction peaks *w* (101) and *w* (102) at 2θ = 27.33° and 30.53°, respectively. These findings align with previous study [21]. Conversely, as shown by Sample #3 patterns, the weak shoulder on the right side of the *s* (111) peak corresponds to the diffraction of the *w* (102) plane, which indicates the presence of some hexagonality [22,23].

Ueno et al. [24] reported the evolution of the unit cell parameter of Ga-bearing sphalerite as a function of the degree of cationic substitution,

(1) $$R=Ga/\left(Ga+Zn\right)=2x/\left(2-x\right)$$

assuming the following substitutional mechanism:

(2) $$\left(1-\frac{3x}{2}\right)ZnS+\frac{x}{2}{Ga}_{2}S_{3}\to \;{Zn}_{\left(1-\frac{3x}{2}\right)}{Ga}_{x}V_{{Zn}_{\frac{x}{2}}}^{\prime \prime }S\;$$

where *x* designates the cation site fraction occupied by gallium. Our prior study has confirmed Ueno’s findings and shown a shrinkage in lattice constant with increasing Ga concentration [25]. Table 1 summarizes the lattice parameters, phase content, and Ga content of the samples investigated here, as determined by XRD analysis.

**Figure 4:**
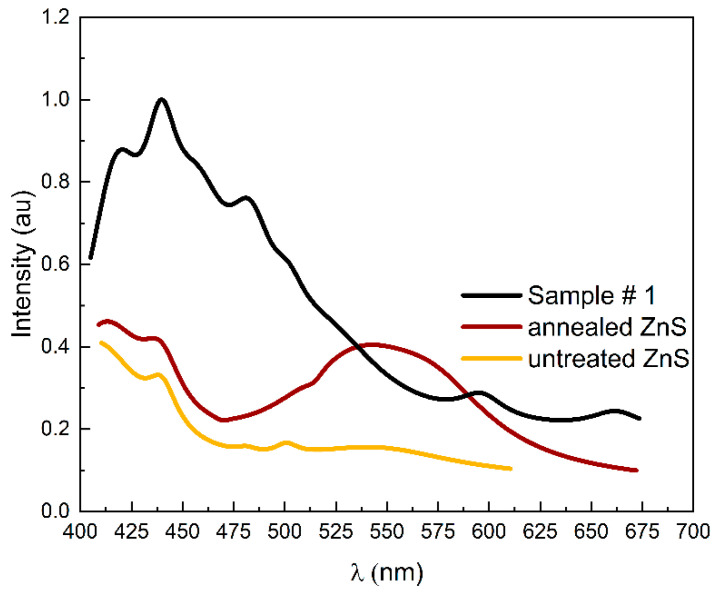
Photoluminescence spectra of untreated MS-ZnS, annealed MS-ZnS, and Sample #1 for 365 nm excitation.

To further elucidate the mechanism of substitution, the PL spectra of untreated, annealed, and Sample #1 are compared in Figure 4. The peaks were adjusted through Lorentz deconvolution. The spectral profiles display distinct shapes, with their respective peaks showing varying positions and levels of intensity. All spectra exhibit a prominent peak at approximately 440 nm that can be attributed to the recombination of electron donors at sulfur vacancies with holes trapped at Zn vacancies [26]. The spectrum of annealed MS-ZnS shares similar spectral features with untreated MS-ZnS, with the exception of a broad band near 550 nm, which can be associated with structural defects resulting from annealing [27]. Interestingly, Sample #1 spectrum reveals two new bands centered at 475 nm and 610 nm, respectively, that are not observed in the annealed MS-ZnS spectrum despite both samples undergoing identical heating and cooling processes. These bands indicate the presence of Ga in ZnS [28,29] and have been linked to the association of ${Ga}_{Zn}^{•}$ and $V_{Zn}^{”}$ to form ${{(V}_{Zn}{Ga}_{Zn})}^{\prime }$ centers (for 475 nm) and ${{(V}_{Zn}{(Ga}_{Zn})}_{2}$ complexes (for 610 nm) [28,30].

### 3.2. Transmittance in IR

**Figure 5:**
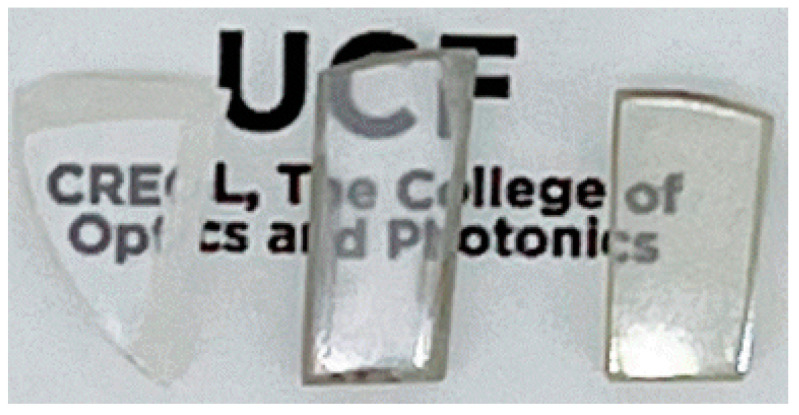
From left to right: untreated, annealed, and Sample #3.

**Figure 6:**
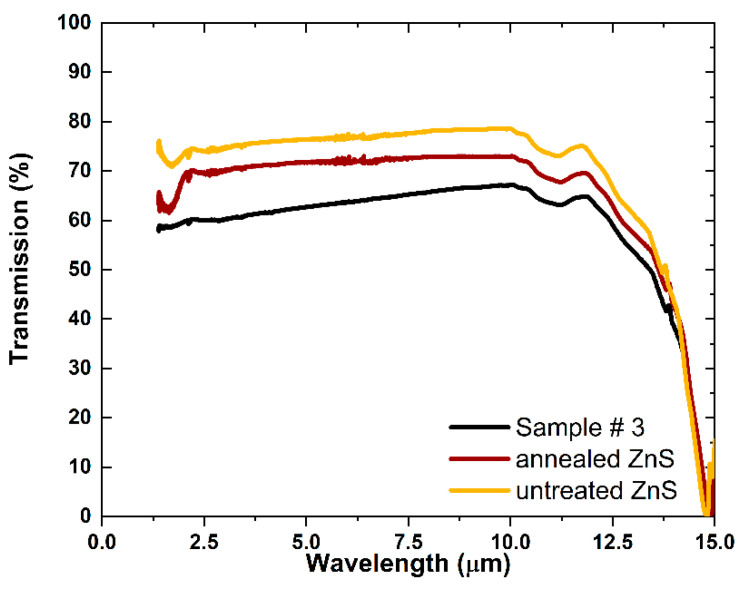
Infrared transmission spectra of untreated MS-ZnS (yellow), annealed MS-ZnS (red), and Sample #3 (black). The thicknesses of these samples are similar, measuring 2.49, 2.44, and 2.40 mm, respectively.

The untreated MS-ZnS, annealed MS-ZnS, and Sample #3 are transparent (Figure 5). Additionally, the infrared transmission of the annealed MS-ZnS is similar to that of untreated MS-ZnS (Figure 6) with a slight decrease that can be attributed to minor differences in the surface finish [23], whereas Sample #3 exhibits further loss due to light scattering. A similar trend was observed by Dunn et al. when co-sintering ZnS with Ga_2_S_3_ [14]. No absorption band is introduced by annealing nor after Ga diffusion. The non-uniform distribution of Ga in both radial and cross-sectional directions, as evidenced by XRD and SIMS analyses, is likely the primary cause of scattering. This phenomenon occurs due to spatial variations in the refractive index at the scale of the wavelength. Additionally, potential differences in lattice and grain boundary diffusion coefficients for Ga in ZnS may contribute to scattering at the grain level.

### 3.3. Hardness

Hardness quantifies the resistance of a material to localized plastic deformation under a fixed load and is often used as an estimate of mechanical strength. The degree of plastic deformation in polycrystalline materials varies depending on factors such as the sliding direction, dislocation density, and the ratio of grain size to indenter size [31,32]. In this study, microhardness measurements were performed under typical conditions to measure the indentation diagonals [33] as accurately as possible and were averaged over 100 locations on the sample to improve the statistics. Additionally, tests that generated cracks (fractures) were discarded from the analysis.

**Figure 7:**
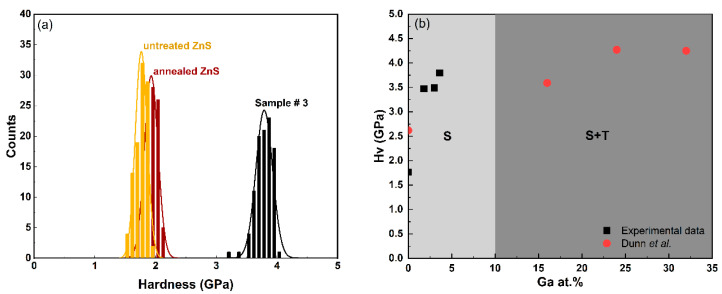
(**a**) Vickers microhardness distribution of untreated MS-ZnS, annealed MS-ZnS, and Sample #3. (**b**) Average microhardness values of Samples #1, #2, and #3 obtained by Ga_2_S_3_-treatement (this work) compared to co-sintered Ga_2_S_3_-ZnS ceramics (from [14]). The single sphalerite (*S*) phase and two-phase sphalerite + tetragonal (*S + T*) domains are both represented in this figure to highlight the difference in hardness improvement one can obtain from cationic substitution (single-phase solute strengthening) and precipitation hardening (presence of two-phases). The latter comes at the expense of transparency due to scattering.

**Figure 8:**
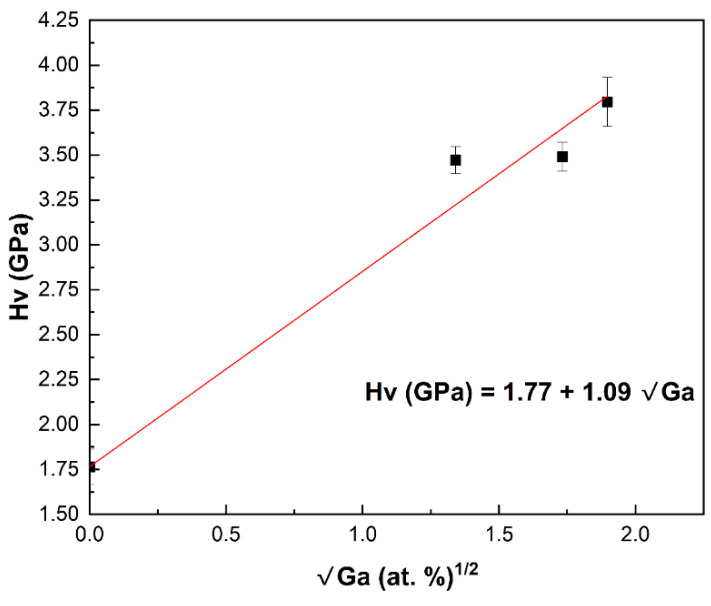
Linear evolution of hardness with $\sqrt{Ga}$ concentration in Ga_2_S_3_-treated MS-ZnS.

The histogram in Figure 7a shows the results of one hundred Vickers microhardness tests conducted on each sample. The data indicate that untreated and annealed MS-ZnS have comparable hardness, with overlapping distributions and no notable differences in the average microhardness values or standard deviations. In contrast, Sample #3 demonstrates enhanced hardness, reaching up to 2.2 times that of untreated MS-ZnS. A comparison between our experimental data and Dunn’s group findings is presented in Figure 7b. Samples #1, #2, and #3 compositions are within the sphalerite-pure region, suggesting that the observed strengthening in these specimens is attributable to solute strengthening despite the presence of hexagonality or wurtzite as revealed by XRD analysis in (Figure 3). This implies that Ga may have initiated the nucleation of a small amount of wurtzite, insufficient to impose a hardening mechanism. The FTIR results further support this conclusion. This outcome differs from Dunn et al.’s experimental data [14], where strengthening results from zinc thiogallate (ZnGa_2_S_4_) precipitation, as all sample compositions fall within the two-phase region where sphalerite and tetragonal phases coexist. The significance of our work lies in demonstrating that diffusing Ga into the MS-ZnS matrix has a substantial hardening effect while maintaining a single, optically transparent, sphalerite phase. Additionally, Figure 8, which graphically presents the data from Table 2, illustrates an important finding: The microhardness linearly increases with the square root of Ga concentration, aligning with Fleisher’s mechanism. This observation is consistent with several previous studies [34,35,36] that demonstrated the proportionality between the stress field intensity in slip planes and solute concentration, which impedes dislocation motion [35], thereby strengthening the material.

## 4. Conclusions

Diffusing Ga_2_S_3_ into multispectral-grade ZnS by thermal vapor deposition resulted in substantial improvements in surface microhardness, with values more than doubling compared to untreated windows (3.80 GPa for 3.6 at% Ga-doping versus 1.77 GPa for untreated window). The observed linear increase in hardness with the square root of Ga concentration aligns with Fleisher’s mechanism, indicating solute strengthening as the primary hardening mechanism. Importantly, this hardening is achieved without inducing significant phase changes, thus preserving broadband transmission and minimizing infrared transmission loss. Although promising, this approach will require further investigation to assess microstructural changes, long-term mechanical durability, and optical performance under operational conditions. Additionally, optimizing the diffusion process will be essential to ensure uniform strengthening, especially in larger optics.

## Figures and Tables

**Table 1:** Lattice parameter, phase wt. %, and Ga content of untreated MS-ZnS, annealed MS-ZnS, and Ga_2_S_3_-treated MS-ZnS.

	Untreated MS-ZnS	Annealed MS-ZnS	Ga_2_S_3_-Treated MS-ZnS		
			Sample 1	Sample 2	Sample 3
Lattice parameter, (Å)	5.411(0)	5.411(0)	5.406(9)	5.405(1)	5.404(3)
Sphalerite phase, (wt. %)	100	100	~100	~100	~100
Wurtzite phase, (wt. %)	0	0	0	0	<0.1
Gallium content, x, (at. %)	0	0	1.8	3.0	3.6

**Table 2:** Results of Vickers microhardness indentation on MS-ZnS, annealed, and of Samples #1, #2, and #3. The data represent the average value (H_v_) and standard deviation (σ_Hv_) for 100 tests on each sample.

Ga (at. %)	H_v_ (GPa)	σ_Hv_ (GPa)
0 (untreated)	1.77	0.10
0 (annealed)	1.92	0.11
1.8	3.47	0.08
3.0	3.49	0.08
3.6	3.80	0.14

**Figure A1:**
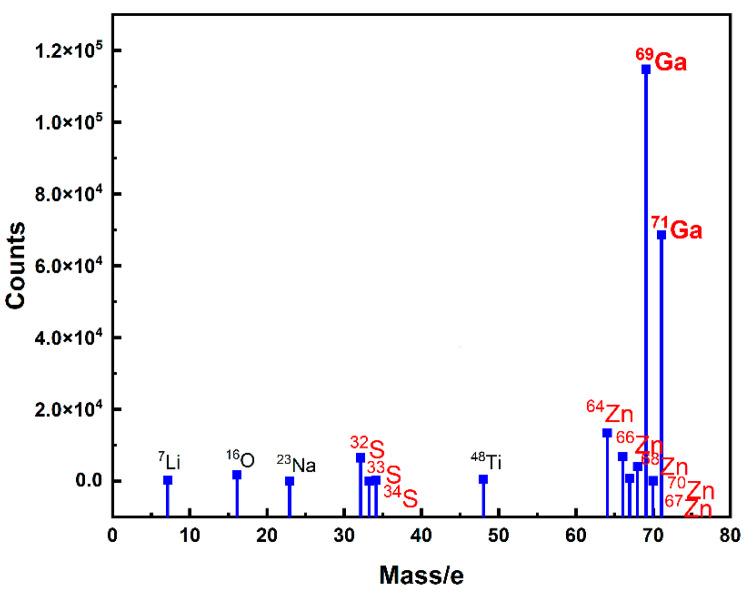
SIMS spectrum Sample #3 collected from the surface.

**Figure A2:**
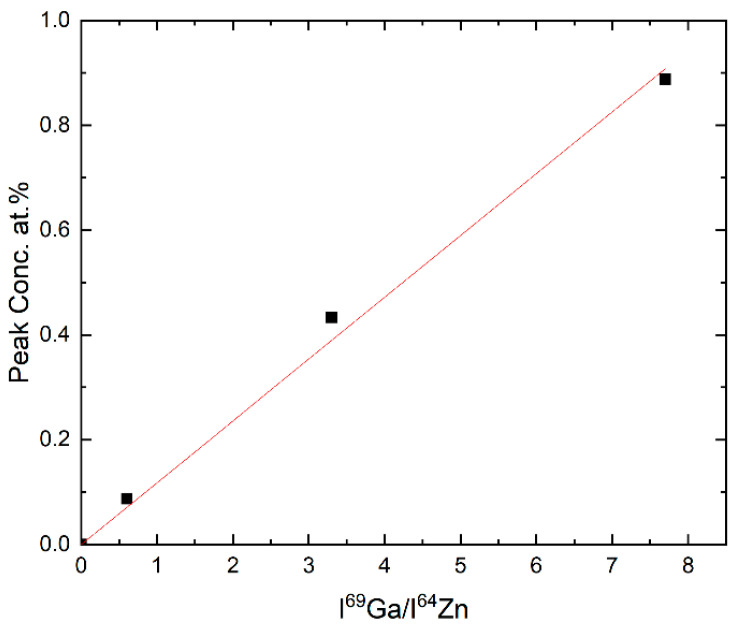
Calibration curve of ^69^Ga concentration versus the experimental SIMS intensity ratio I^69^Ga/I^64^Zn.

**Figure A3:**
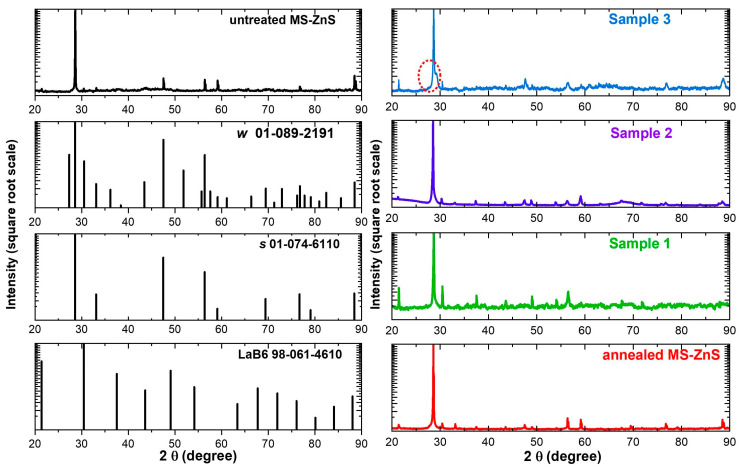
From bottom to top: X-ray diffraction patterns of LaB_6_, *s*, and *w* ZnS (as per reference card #98-061-4610, #01-074-6110, and #01-089-2191, respectively), and untreated ZnS (**left**). From bottom to top: X-ray diffraction patterns of annealed ZnS, Samples #1, #2, and #3 (**right**). The red circle shown on the pattern of sample #3 highlights the broadening of the *s* (111) due some hexagonality.

## Data Availability

The original contributions presented in this study are included in the article. Further inquiries can be directed to the corresponding author.

## Appendix A

### Appendix A.1. SIMS Data Analysis

Figure A1 shows the SIMS spectrum of Sample #3 measured on its surface. The peaks of ^69^Ga and ^71^Ga appear very intense, clearly showing that gallium sulfide was deposited on the surface of the sample.

RSF values for ^69^Ga in ZnS were calculated using an integration approach similar to [37] (Table A1). RSF converts the count rates to atom density [38,39,40] (Equation (A1)):

(A1) $$C_{69_{Ga}}\;\left(at.\%\right)=\frac{1}{\rho }\;\frac{I_{69_{Ga}}}{I_{64_{Zn}}}\times RSF\;\left(at\cdot {cm}^{-3}\right)\times 100$$

Here, the factor 1/*ρ* allows for the conversion of concentrations in (at·cm^−3^) to (at. %). The experimental density of pure MS-ZnS $\rho =5.05248\times 10^{22}\;(at\cdot {cm}^{-3}$) was adopted by considering both the standards and all samples in this study as dilute solid solutions [38,39,40]. Then, knowing the concentration of ^69^Ga in the standards and selecting ^64^Zn as the matrix major isotope, a calibration curve of concentration versus normalized ^69^Ga intensity ($\frac{I_{69_{Ga}}}{I_{64_{Zn}}}$) can be established and fitted to a linear model (Figure A2).

(A2) $$C_{69_{Ga}}\;\left(at.\%\right)=0.118\times \frac{I_{69_{Ga}}}{I_{64_{Zn}}}$$

Using the SIMS results of the Ga_2_S_3_-treated samples, the concentration of ^69^Ga is deduced according to Equation (A2) and converted into the total gallium content by taking the natural abundances of ^69^Ga, $A_{69Ga}$= 60.10% [41] into account:

(A3) $$C_{Ga}\;\left(at.\%\right)=\frac{C_{69_{Ga}}\;\left(at.\%\right)}{A_{69Ga}}$$

**Table A1:** RSF values of ^69^Ga in ZnS over the concentration range 0.1 to 1 (at. %).

^69^Ga-Implanted ZnS Standards (at. %)	0.1	0.5	1.0
RSF $\left(at\cdot {cm}^{-3}\right)$	7.52 × 10^19^	6.68 × 10^19^	5.76 × 10^19^

### Appendix A.2. XRD Data

The X-ray diffraction patterns of all samples are presented in Figure A3. The square root of the intensities was plotted against 2θ to make peaks with smaller intensities more visible. X-ray diffraction patterns of LaB_6_, *s*, and *w* ZnS are included for comparison.
